# Chicago Medical Examiner

**Published:** 1860-01

**Authors:** 


CHICAGO MEDICAL EXAMINER.—This is the name of a new medical periodical, just established by Dr. N. S. Davis, Professor in the Lind University, at Chicago, and Dr. E. A. Steele. It is to be issued monthly, and is to be devoted to the educational, scientific, and practical interests of the profession.

